# Validation of p53 Immunohistochemistry (PAb240 Clone) in Canine Tumors with Next-Generation Sequencing (NGS) Analysis

**DOI:** 10.3390/ani13050899

**Published:** 2023-03-01

**Authors:** Barbara Brunetti, Dario de Biase, Giulia Dellapina, Luisa Vera Muscatello, Francesco Ingravalle, Giorgia Tura, Barbara Bacci

**Affiliations:** 1 Department of Veterinary Medical Sciences, University of Bologna, 40126 Bologna, Italy; 2 Department of Pharmacy and Biotechnology, University of Bologna, 40126 Bologna, Italy; 3 Biostatistics, Epidemiology and Risk Analysis (BEAR), Istituto Zooprofilattico Sperimentale del Piemonte, Liguria and Valle d’Aosta, 10154 Turin, Italy

**Keywords:** accuracy, canine tumors, immunohistochemistry, NGS, p53, sensitivity, specificity, *TP53*, tumors

## Abstract

**Simple Summary:**

*TP53* is a tumor-suppressor gene that is involved in cell cycle arrest, and its mutation is an event that is recognized to cause and contribute to carcinogenesis. Increased immunohistochemical expression of p53 in tumors has been associated with mutation of the gene; therefore, this can be an important tool to detect p53 anomalies. In this study, the expression of p53 was compared to NGS analysis in order to verify if p53 could predict *TP53* gene mutation. The results indicate that with a 10% threshold, the sensitivity of immunohistochemistry was 60%, the specificity was 86.7%, and the accuracy was 76%. This suggests that we can expect up to 25% inaccurate predictions. Moreover, a substantial decrease in sensitivity was observed, moving the threshold to 50%. Overall, these results suggest that IHC can predict mutation and that the 10% threshold can be considered appropriate.

**Abstract:**

In human medicine, p53 immunohistochemistry (IHC) is a common method that is used for the identification of tumors with *TP53* mutations. In veterinary medicine, several studies have performed IHC for p53 in canine tumors, but it is not known how well it actually predicts the mutation. The aim of this study was to estimate the accuracy of the IHC method for p53 (clone PAb240) using a lab-developed NGS panel to analyze *TP53* mutations in a subset of malignant tumors in dogs. A total of 176 tumors were analyzed with IHC and then 41 were subjected to NGS analysis; among them, 15 were IHC positive and 26 were negative, and 16 out of 41 (39%) were found to be inadequate for NGS analysis. Excluding the non-evaluable cases at NGS, of the remaining eight IHC-positive cases, six were mutants and two were wild-type. Among the 17 IHC-negative cases, 13 were wild type, and 4 were mutants. The sensitivity was 60%, specificity was 86.7%, and the accuracy was 76%. These results suggest that when using IHC for p53 with this specific antibody to predict mutation, up to 25% wrong predictions can be expected.

## 1. Introduction

*TP53* is a well-known tumor suppressor gene that encodes the p53 protein, which is involved in cell cycle arrest in damaged cells that require DNA repair or, in cases of damage beyond repair, triggering apoptosis. Immunohistochemical detection of p53 is used in daily practice as a surrogate marker of mutation in various human cancers [1]. However, there are conflicting opinions regarding the reliability of IHC to detect p53 anomalies. Mutant p53 protein has a significantly longer half-life and greater stability than its wild-type counterpart and is, therefore, detectable by immunohistochemical methods [2]. It is reported that missense mutations lead to the production of a non-functional protein with a longer life span and is thus detected by IHC as strong and diffuse nuclear staining [3,4]. In humans, it is reported that the majority of *TP53* mutations are missense substitutions (75%) [5]. On the contrary, there are null mutations or non-sense mutations that lead to no p53 protein expression at all, and this is reflected by a complete absence of immunostaining [3,6,7]. Moreover, it is now known that certain types of mutations, such as truncating mutations, do not result in protein stabilization [7]. Furthermore, it appears that wild-type p*53* can also be stabilized in the absence of mutations, for example, with heat, oxidative stress, irradiation, or chemotherapeutic agents [8,9]. Nevertheless, many studies in human medicine have validated the use of IHC for p53 to identify cases with mutations of the *TP53* gene [1,10,11]. Therefore, while IHC is a useful, cost-effective, and rapid tool to detect p53 accumulation, further analysis by gene sequencing is needed to confirm the mutational status in canine species and to correlate it with the biological behavior and prognosis of tumors.

In the canine species, for which many studies are based on immunohistochemistry alone, altered p53 immunoexpression has been reported in different tumors, such as cutaneous hemangiosarcomas [12,13], mammary carcinomas [13,14,15], osteosarcomas [2,16,17,18], liposarcomas [19], mast cell tumors [20], squamous cell carcinomas [21], transmissible venereal tumors [22], prostate cancer [23], and epithelial colorectal tumors [24]. Different monoclonal and polyclonal antibodies were used in these studies, and they had conflicting results in terms of both the extent of positivity and the correlation with prognosis. In a study by Zacchetti et al. (2007) on canine tumors, the authors compared the immunohistochemical expression of p53 with its mutational status in 6 sarcomas and 21 mammary carcinomas using six anti-p53 antibodies, then they compared the results with those of DNA analysis. Only the monoclonal PAb240 and PAb421 and the polyclonal CM1 antibodies were able to detect the expression of the canine p53 protein. CM1 had the highest concordance between positive expression of p53 protein by IHC and presence of a gene mutation, while the concordance of PAb240 was lower [25].

Generally, in both human and veterinary medicine [14,19,26], a 10% cutoff for nuclear immunopositivity is used, but as there is disagreement among authors, where several cutoff values have been used for both humans [1,7] and dogs [12,15,18,27].

Next-generation sequencing (NGS) can be used to detect *TP53* mutations, but this technique has a high working load and high costs. These disadvantages are compensated by the possibility of detecting a wide range of mutations and the high sensitivity of the technique, but due to its high cost, it cannot be used for routine analysis in veterinary medicine.

Hence, the aim of the present study was to measure the accuracy of the immunohistochemical method for p53 using NGS (used as a gold standard) to analyze *TP53* mutations in different types of canine tumors that were previously tested with IHC with the monoclonal antibody PAb240. Another aim of the work was to apply two IHC positivity cutoff values to identify which one would correlate better with NGS results.

## 2. Materials and Methods

### 2.1. Case Selection, Histological Classification, and Grading

The study was based on 176 canine tumors that were identified in the archives of the Anatomic Pathology Section of the Department of Veterinary Medical Sciences of the University of Bologna and the Anicura Veterinary Hospital “I Portoni Rossi”, Bologna, Italy, for which formalin-fixed paraffin-embedded (FFPE) specimens were available. Samples included 89 mammary carcinomas, 23 squamous cell carcinomas (SCCs), 19 soft tissue sarcomas (STSs), 19 amelanotic melanomas from skin and oral mucosa, 22 mast cell tumors, and 4 osteosarcomas. Whenever possible, we selected cases with the highest histological grades.

Hematoxylin and eosin histological sections were reviewed by 2 board-certified authors (BBr, LM) until a consensus diagnosis was reached, and cases were classified according to the most recent classification [28,29,30] and histological grading [31,32,33].

### 2.2. Immunohistochemistry

Immunohistochemistry for p53 was performed in each case on 3 µm thick slides following a previously described method using a monoclonal antibody (clone PAb240, 1:200 dilution; BD Pharmigen). Cases were considered positive if >10% of the neoplastic cells displayed nuclear positivity, and cases with >50% neoplastic positive cells were recorded. Sections of canine mammary carcinoma in which the expression of p53 was known were used as positive controls. The PAb240 clone recognizes both wild-type and mutated *TP53*, and its cross-reactivity with canine tissues has already been demonstrated [34,35].

### 2.3. Next-Generation Sequencing (NGS)

All immune-positive cases and 26 negative cases were analyzed with NGS. Sequencing with NGS was performed at the Molecular Pathology Laboratory of the Policlinico Sant’Orsola-Malpighi, Bologna, Italy.

For each paraffin block, only tumor areas with a greater tumor cellular component were selected, avoiding non-tumor areas, necrotic areas, and areas rich in stroma. This selection was made by observing the slide under a microscope and selecting the areas to be removed from the paraffin block with a marker. Obviously in cases such as mammary comedocarcinoma in which the necrotic areas are closely associated with the tumor parenchyma, this was note possible. DNA was extracted from two to three 10 µm thick tissue sections, which were scratched manually with a sterile blade from two or three sections, depending on the amount of neoplastic tissue present. DNA extraction was performed using 50–70 µL of Quick Extract FFPE Kit (Lucigen, LGC Biosearch Technologies, Hoddesdon, UK) in order to allow the melting of paraffin and the degradation of cellular components. DNA was quantified using a Qubit 1.0 fluorometer (Thermo Fisher Scientific, Waltham, MA, USA), according to the manufacturer’s recommendations.

DNA was amplified using a laboratory-developed NGS panel (Molecular Pathology Laboratory of the Policlinico Sant’Orsola-Malpighi). The panel was designed using the Ion AmpliSeq Designer Tool (Thermo Fisher Scientific) and allowed amplification and sequencing of all the coding sequences (CDSs) of the *TP53* gene (start chr5:32560598, end chr5:32565670; reference: *Canis lupus familiaris 3*). The panel included a total of 48 amplicons between 125 and 175 bp in length. A total of 30–40 ng of DNA was used for library amplification, using the AmpliSeq Plus Library Kit 2.0 (Thermo Fisher Scientific, Waltham, MA, USA).

Briefly, 2 pools of the same sample were merged together and 2 µL of FUPA enzyme was added. At the end of the digestion step, 4 µL of Switch Solution, 2 µL of IonCode, and 2 µL of DNA ligase were added.

A total of 45 µL of magnetic beads (AMPure, Beckman Coulter, Brea, CA, USA) were added to 30 µL of indexed libraries (beads/libraries = 1.5). The samples were then incubated on a magnetic plate for 5 min and washed with 150 µL of 70% ethanol. Finally, the samples were eluted with 50 µL of tris-EDTA.

Quantification of the libraries was carried out by real-time PCR using a Quantitative IonQuant kit (Thermo Fisher Scientific, Waltham, MA, USA). The calibration curve was generated using *E. coli* DNA at concentrations of 6.8, 0.68, and 0.068 ng/µL. Amplicon libraries were diluted to 1:100 and quantified using the Ion TaqMan^®^ Assay (Thermo Fisher Scientific, Waltham, MA, USA). Finally, the samples were diluted to a final concentration of 22 pM and loaded on the IonChef machine in a single pool for the emulsion PCR step.

The sequencing was performed using the GeneStudio S5 Sequencer (Ion 530^TM^ Chip) and the results were analyzed using Integrated Genome Viewer software (IGV v2.9.2, http://software.broadinstitute.org/software/igv/). To avoid false positive results, only the nucleotide variants with a variant allele frequency (VAF) higher than 10% of the total number of analyzed sequences were used for the mutational call. The functional interpretation of the mutation was evaluated using the PolyPhen2 tool (http://genetics.bwh.harvard.edu/pph2/) PolyPhen-2 score is a tool which predicts the possible impact of an amino acid substitution on the structure and function of a protein.

### 2.4. Statistics

Accuracy was evaluated based on sensitivity, specificity, and predictive values using diagnostic testing accuracy [36].

The accuracy of IHC compared to NGS was evaluated by calculating the sensitivity, specificity, and predictive value (and their 95% confidence interval (CI) [37]. When a mutation of *TP53* was detected, samples were considered true positives; on the contrary, wild-type *TP53* samples were considered true negatives. Therefore, IHC-positive samples with wild-type *TP53* were considered false positives, and samples with *TP53* mutation and negative IHC were considered false negatives.

Furthermore, differences in the percentage of immunoreactivity in IHC between wild-type and mutated TP53 were investigated with the Mann–Whitney test and by fitting an ordered logistic model. IHC immunoreactivity was evaluated based on 3 classes: <10%, ≥10–50%, and ≥50%.

*p*-values < 0.05 were considered significant. Statistical analyses were carried out using STATA 17.0 statistical software (StataCorp, College Station, TX, USA).

## 3. Results

### 3.1. Histopathology and Grading

A total of 89 cases of mammary carcinomas were classified as follows: 15 tubular carcinomas, 11 papillary carcinomas, 11 tubulo-papillary carcinomas, 18 solid carcinomas, 6 comedocarcinomas, 9 complex carcinomas, 5 ductal carcinomas, 6 mixed carcinomas, 1 adenosquamous carcinoma, 2 micropapillary carcinomas, and 5 intraductal carcinomas. Among them, 28.09% were Grade 1, 32.58% were Grade 2, and 31.46% were Grade 3.

Among the 19 soft tissue sarcomas, 11 (57.89%) were Grade 2 and 8 (42.10%) were Grade 3. Among the 22 mast cell tumors, 5 were high grade (22.72%) and 17 were low grade (77.28%). The 23 squamous cell carcinomas, 19 amelanotic melanomas, and 4 osteosarcomas were not graded.

### 3.2. Immunohistochemistry

Of the 176 canine tumors that were examined, only 15 (8.52%) were positive for p53 by immunohistochemistry.

**Figure 1:**
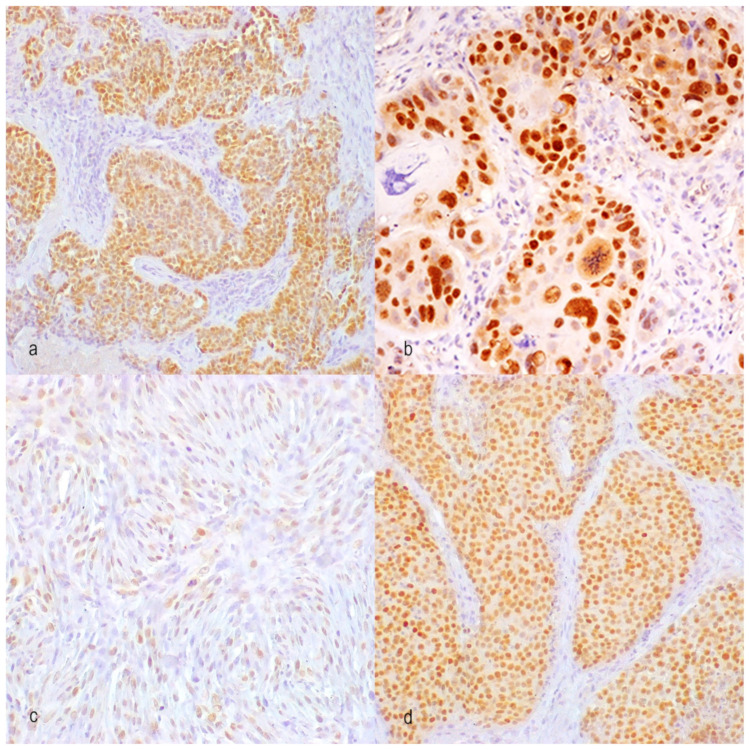
Immunohistochemistry of dog anti-p53 antibody. (**a**) Mammary carcinoma; the majority of the nuclei were immunolabeled (4×). (**b**) Squamous cell carcinoma; the nuclei were strongly and diffusely immune-positive (20×). (**c**) Soft tissue sarcoma; about half the cells had an immune-positive nucleus (10×). (**d**) Amelanotic melanoma; all nuclei of tumor were immunolabeled (10×).

The three positive cases were mammary carcinomas (3.37% of 89 cases), of which one was a solid Grade 2 carcinoma, and two were Grade 3 comedocarcinomas (Figure 1a). A total of six positive cases were squamous cell carcinomas (26.08% of 23 cases) (Figure 1b), and three cases were soft tissue sarcomas (15.78% of 19 cases) (Figure 1c), of which two cases were Grade 2 and one was Grade 3. There were two oral amelanotic melanomas (10.52% of 19) (Figure 1d) and one high-grade mast cell tumor (4.54% of 22 cases) that were positive. Osteosarcomas were all negative. Of the total positive cases, 11 had more than 50% positive cells and 4 had 10–50% (Table 1).

### 3.3. Next-Generation Sequencing (NGS)

For the following scientific work, only the p53 panel was considered and was designed for all *TP53* CDS from exon 1 to 10, therefore, it was expected to detect almost all possible protein domains. Exons 9 and 10 have lower coverage (less than 150×) in terms of “reads”, therefore, any mutations on these exons may be undetectable. For the sequencing, 15 IHC-positive samples were selected and 7 of them (46.66%) were found to be non-evaluable due to high DNA degradation. Of the immune-negative cases, 9 out of 26 (34.6%) were non-evaluable. The non-evaluable (inadequate) cases were mostly mammary (10/18, 55.55%) and cutaneous (3/14, 21.42%). Following the analysis, two samples were wild-type (2/8, 25% false positives), while at least one mutation was identified in the remaining six samples (6/8, 75% true positives). Among the six mutated cases, three had single mutations, one had two mutations, and two had three p53 mutations. There were ten different types of mutations that were found; all were missense mutations except two cases, which had an identical non-sense mutation (Table 1).

Furthermore, 26 IHC-negative cases were randomly selected among the tumors with the highest histological grade. A total of nine of these were non-evaluable (9/26, 34.6%). Among the remaining 17 samples, 13 were wild-type (76.47%) (Supplementary Table S1) and 4 had single mutations (23.52%), specifically 3 missense and 1 non-sense mutation (Table 2). The non-sense mutation was the same as the two true positive cases with non-sense mutation.

### 3.4. Statistics

**Scheme 1:**
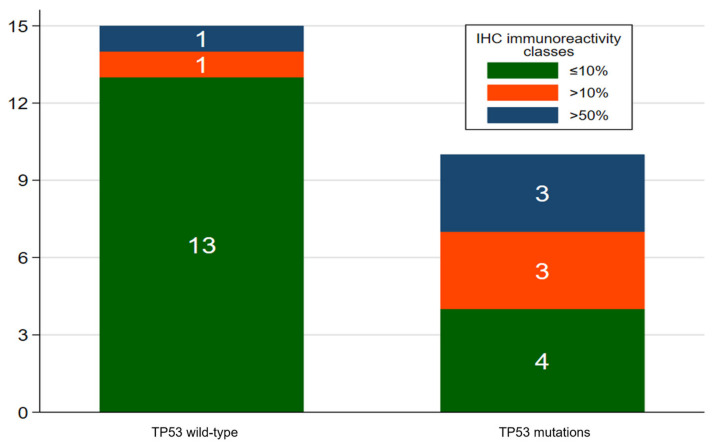
Cases evaluated with IHC and NGS, considering p53 immunopositivity threshold equal or greater than 10%.

The accuracy results are reported in Table 3 and Table 4 and Scheme 1. Table 3 and Graph 1 show the results of p53 immunopositivity at values equal to or greater than 10%, while Table 4 shows the results that were obtained by setting the immunopositivity threshold to values equal or greater than 50%.

The Mann–Whitney test results suggest a significant difference for IHC-positive cell classes between wild-type samples and samples with mutations (*p*-value = 0.0354).

Furthermore, the ordered logistic model suggests an effect of the presence of mutations on the IHC-positive cell classes (OR = 8.9, 95%CI: 1.3–60.2) and a larger probability of mutation in IHC classes with immunoreactivity ≥10% than in classes with immunoreactivity <10%.

## 4. Discussion

*TP53* is the most frequently mutated gene in human cancers, and its mutation affects about 50% of human malignancies, mainly in the form of missense mutations [38,39]. In our series, the cases that were positive on immunohistochemistry accounted for 8.52% of the total number of analyzed cases. Based on tumor types, 3.37% of p53-positive cases were in mammary carcinomas, 26.08% in squamous cell carcinomas, 15.78% in soft tissue sarcomas, 10.52% in amelanotic melanomas, and 4.54% in mast cell tumors. As expected, these percentages are lower compared with data using a polyclonal antibody [2,21,40]. In fact, previous studies that used polyclonal CM-1 antibody showed positivity in 22% of canine lymphomas [26], 37.5% of squamous cell carcinomas [21], and 44% of colorectal epithelial tumors [24]. By contrast, immunohistochemical analysis with the polyclonal FL-393 antibody on canine cutaneous mast cell tumors showed only 17/71 p53-positive cases (23.94%). Studies in which monoclonal antibodies were used had lower positivity percentages, as these antibodies are more specific but less sensitive. In our previous study on 170 canine mammary carcinomas with PAb240, we found 4.7% positivity [14]; to confirm these data, in the present work on a different series of canine mammary carcinomas, we had similar positivity of 3.37%. A similar study on mammary carcinoma found 7 out of 35 positive cases (20%) [15].

The polyclonal CM-1 antibody has also been employed in canine squamous cell carcinomas, showing p53 expression in 37.5% of cases [21], higher than the 26.08% in the present study. The lower percentages in our study may be due to different reasons: the use of different antibodies (monoclonal or polyclonal), the scoring method, or the pre-analysis variables. To date, only a few studies have compared the concordance between different clones of p53 antibodies and the presence of mutations [25,34,35]. In a study by Zacchetti et al. (2007) [25], six monoclonal antibodies and one polyclonal antibody were tested in six sarcomas and 21 mammary carcinomas from 27 dogs, and the results were compared with the gene mutation analysis. The monoclonal PAb240 antibody was able to detect the expression of canine p53 protein with gene mutation with a concordance of 45.45% (5/11). As previously mentioned, CM1, which is polyclonal, was found to give the highest concordance (8/11). Currently, in human medicine there is no consensus as to which antibodies are most appropriate for evaluating mutation-associated p53 expression.

In our study, the positivity of a small number of tumors allowed subsequent NGS analysis of a small number of cases. This was mostly due to the high degradation of FFPE tissues, which resulted in 16 inadequate samples (39% of cases sequenced). This obstacle is acknowledged in an article by Ihle et al. (2014) [41], which noted that one of the difficulties of PCR-based amplification methods is the fixation artifacts of FFPE samples. These artifacts seem to be due to post-mortem phenomena, including the deamination of cytosines and adenines, which result in uracil residues and hypoxanthine, respectively. The DNA contained in formalin-fixed tissue undergoes alterations with chemical modification and fragmentation, and this makes it difficult to obtain samples that are suitable for molecular analysis.

Following NGS analysis, we compared the NGS and IHC methods applying a ≥10% positivity threshold, and calculated a sensitivity of 60% and a specificity of 86.7%, with a total accuracy of 76%. These values are similar to those that were reported in human medicine, with concordance between p53 IHC and *TP53* mutation status ranging from 55 to 89% in human gliomas (Grade I-IV) [42]. Moreover, in the same studies, the false-positive rate (the incidence of p53 IHC positivity with the presence of wild-type *TP53*) ranged from 2 to 45%. One study on human glioblastomas found 66% true positives, which are similar to the results of the present study [42]. In addition, another study found 6026/7878 IHC-positive cases (76.5%) with *TP53* mutations in different human cancers [43].

As shown in Table 3, the positive predictive value was 75% (95%CI 34.9–96.8%) using a ≥10% threshold for p53 IHC positivity, with a corresponding false positive rate of 25%. This percentage can be explained by the antibody clone (PAb240), which does not discriminate between wild-type and mutant p53 proteins. In fact, false positivity occurs in cases where wild-type *TP53* is either overexpressed or exhibits a prolonged half-life. There are several recognized causes of half-life prolongation, such as heat, oxidative stress, irradiation, and chemotherapeutic agents [8,9]. The two false positive cases were an SCC (10–50% positivity) and an STS (≥50% positivity). The 50% threshold was applied to verify whether a higher threshold would prevent false positives, but in one case >50% positivity corresponded to wild-type *TP53*. Mutations were also found in cases that had 10 to 50% positivity, confirming that the 10% cutoff value is reliable. Using a ≥50% threshold compared to ≥10% does not seem to provide any benefits; in fact, the sensitivity drops by half (30%) even if the PPV is unchanged, the specificity is significantly increased (93%), the NPV drops by 10%, and the accuracy drops by 8%. For these results, the threshold of ≥10% is, therefore, considered to be more accurate than ≥50%. It is important to note that with the latter threshold, accuracy was moderate (76%); in fact, 25% false positives and false negatives is not a negligible proportion. On the other hand, the Mann–Whitney test and the ordered logistic model suggest that the higher the IHC immunoreactivity percentage, the greater the probability of mutation. Since IHC immunoreactivity was <10% for most cases (15 out of the 25 included in this statistical analysis), a more-in-depth IHC study using a larger case series with a ≥10% threshold would be appropriate. The major limitation of the study was the small number of cases that were analyzed; however, it must be noted that the initial number of cases was extremely high, which demonstrates the difficulty of collecting larger amounts of p53-positive tumors. Furthermore, NGS is very expensive and not easily accessible, which are obstacles for current and future studies. Further studies will be needed to truly establish whether IHC for p53 can be a reliable substitute for genetic investigations in canine tumors. In our previous study on canine mammary carcinomas [14] employing the same antibody (Pab240), the few positive cases were in fact related to a worse prognosis.

A total of 13 types of mutations were found in this case series (nine TP, three FN, one in both); most of these were missense mutations in both groups. The PolyPhen2 score, which predicts the possible impact of an amino acid substitution on the structure and function of a human protein, was always 1 or very close to 1 in this study, indicating a high likelihood of damage to the protein. A single FN case had a non-sense mutation with a stop codon that probably generated a truncated protein that could not be detected with immunohistochemistry. As discussed earlier, FN cases may be due to the specific monoclonal antibody, because if the mutations are located outside of its binding site, they will not be recognized. Another explanation for the FN results may be the overexpression of *MDM2* or *CDK2NA* homozygous deletions, which can cause degradation of mutant *TP53*, potentially resulting in a false-negative p53 IHC result [42]. A proportion of FP cases can also be explained by the type of antibody used, as it is known that Pab240 detects both mutant and wild-type p53 protein. In addition, for both FP and FN cases, it must be considered that IHC and NGS are not performed exactly in the same tissue material, hence we assumed molecular homogeneity of *TP53* mutations within a given tumor sample. As such, it is conceivable that mutational variability could exist within different sections of the same tumor specimen.

Finally, the presence of many non-adequate (NE) cases for NGS was due to the inability to extract sufficient DNA from the FFPE material. This may be due to pre-analysis conditions, such as prolonged formalin fixation, which can be a significant issue in veterinary medicine [44,45]. This highlights that sampling and fixation of tumor tissues are of critical importance for both immunohistochemical and molecular techniques.

## 5. Conclusions

In this study, immunoexpression of p53 was compared to NGS analysis in order to verify whether p53 immunopositivity can predict *TP53* gene mutations. The results suggest that when using IHC for p53 with the specific antibody in this study (clone PAb240, BD Pharmigen) to predict mutation, the sensitivity was 60%, specificity was 86.7%, and the accuracy was 76%, which means that up to 25% wrong predictions can be expected. Moving the IHC threshold to ≥50%, the sensitivity was 30%, specificity was 93%, and the accuracy was 68%. Therefore, our results suggest that setting the threshold for the immunohistochemical evaluation of p53 at ≥10% is the most appropriate.

## Figures and Tables

**Table 1:** Tissue, tumor type, histological grade, immunohistochemical positivity, and *TP53* mutation results for each p53-positive case (15/176) are reported. NE, non-evaluable; WT, wild-type; SCC, squamous cell carcinoma; MCT, mast cell tumor; STS, soft tissue sarcoma.

Tissue	Histotype	Grade	IHC Class	TP53 (Ref. TP53-202)	Mutation	PolyPhen2 Score
Mammary (3)	Comedocarcinoma	3	>50%	NE	-	-
	Solid carcinoma	2	10–50%	NE	-	-
	Comedocarcinoma	3	>50%	NE	-	-
Skin (6)	SCC	-	10–50%	p.C290Y	Missense	1
	SCC	-	10–50%	p.Y257F p.R265Stop p.C329F	Missense Non-sense Missense	1 1 1
	SCC	-	>50%	p.P202L p.P203S p.R265Stop	Missense Missense Non-sense	0.969 0.997 1
	SCC	-	>50%	p.P193L p.H230D	Missense Missense	1 0.993
	SCC	-	10–50%	WT	-	
	SCC	-	10–50%	NE	-	
Skin (1)	MCT	high	10–50%	p.R209S	Missense	1
Oral (2)	Amelanotic melanoma	-	>50%	p.G318E	Missense	1
	Amelanotic melanoma	-	10–50%	NE	-	
Subcutis (3)	STS	2	>50%	WT	-	
	STS	2	>50%	NE	-	
	STS	1	10–50%	NE	-	

**Table 2:** p53-negative tumors with <10% positive cells. Only the four cases with *TP53* mutations are reported (4/26). SCC, squamous cell carcinoma.

Tissue	Histotype	Grade	IHC Class	TP53 (Ref. TP53-202)	Mutation	PolyPhen2 Score
Mammary (2/15)	Solid carcinoma	3	<10%	p.C186Y	Missense	1
	Comedocarcinoma	3	<10%	p.P372S	Missense	0.997
Skin (1/5)	SCC	-	<10%	p.R265Stop	Non-sense	1
Bone (1/1)	Osteosarcoma	-	<10%	p.R301P	Missense	1

**Table 3:** Accuracy results and their 95% CI of TP53 genetic sequencing results versus p53 IHC staining results for >10% labelling threshold.

	NGS Mutations	NGS WT	Total	
IHC P53+	6 (TP)	2 (FP)	8	PPV 75% (34.9–96.8%)
IHC P53-	4 (FN)	13 (TN)	17	NPV 76.5% (50.1–93.2.4%)
Total	10	15	25	
	Sensitivity 60% (26.2–87.8%)	Specificity 86.7% (59.5–98.3%)	Accuracy 76% (54.9–90.6%)	

TP, true positive; FP, false positive; FN, false negative; TN, true negative; PPV, positive predictive value; NPV, negative predictive value. Values in parentheses represent lower and upper bounds of 95% confidence.

**Table 4:** Accuracy results and their 95% CI of TP53 genetic sequencing results versus p53 IHC staining results for ≥50% labelling threshold.

	NGS Mutations	NGS WT	Total	
IHC P53+	3 (TP)	1 (FP)	4	PPV 75% (19.4–99.4%)
IHC P53-	7 (FN)	14 (TN)	21	NPV 66% (43–85.4%)
Total	10	15	25	
	Sensitivity 30% (6.7–65.2%)	Specificity 93% (68.1–99.8%)	Accuracy 68% (46.5–85.1%)	

TP, true positive; FP, false positive; FN, false negative; TN, true negative; PPV, positive predictive value; NPV, negative predictive value. Values in parentheses represent lower and upper bounds of 95% confidence.

## Data Availability

Not applicable.

## Supplementary Materials

The following supporting information can be downloaded at: https://www.mdpi.com/article/10.3390/ani13050899/s1, Table S1: List of 26 cases negative to p53 immunoistochemistry, showing that 4/26 cases had TP53 mutation by NGS.
