# Supplementary material for: Validation of p53 Immunohistochemistry (PAb240 Clone) in Canine Tumors with Next-Generation Sequencing (NGS) Analysis
Source: Animals (Basel). 2023 Mar 1;13(5):899. doi: 10.3390/ani13050899 (PMC10000222; doi:10.3390/ani13050899)
Supplement: Supplementary file 1 [file animals-13-00899-s001.zip › animals-2147356-supplementary.pdf]

Supplementary Table S1. List of 26 cases negative to p53 immunoistochemistry, showing that 4/26 cases had TP53 mutation by NGS.

Tissue and histotype	Grade	IHC%	TP53 (Ref.TP53-202)	Mutation	% Mutation	Polyphen2 score
mammary, solid carcinoma	3	<10%	p.C186Y	Missense	25	1
mammary, comedocarcinoma	3	<10%	p.P372S	Missense	10	0.997
mammary, tubular carcinoma	2	<10%	WT			
mammary, solid carcinoma	3	<10%	WT			
mammary, tubular carcinoma	2	<10%	WT			
mammary, papillary carcinoma	3	<10%	WT			
mammary, micropapillary carcinoma	2	<10%	WT			
mammary, papillary carcinoma	2	<10%	WT			
mammary, solid carcinoma	3	<10%	NE			
mammary, solid carcinoms	3	<10%	NE			
mammary, papillary carcinoma	2	<10%	NE			
mammary, solid carcinoma	1	<10%	NE			
mammary, solid carcinoma	2	<10%	NE			
mammary, solid carcinoma	2	<10%	NE			
mammary, micropapillary carcinoma	3	<10%	NE			
skin, SCC	n/a	<10%	p.R265Stop	Non-sense	20	1
skin, SCC	n/a	<10%	WT			

skin, SCC	n/a	<10%	WT			
skin, SCC	n/a	<10%	WT			
skin, SCC	n/a	<10%	NE			
subcutis, STS	2	<10%	WT			
subcutis, STS	2	<10%	WT			
subcutis,STS	2	<10%	WT			
skin, amelanotic melanoma	n/a	<10%	WT			
skin, amelanotic melanoma	n/a	<10%	NE			
bone, osteosarcoma	3	<10%	p.R301P	Missense	75	1

* NE=non evaluable, WT= wild type, SCC=squamous cell carcinoma, STS=soft tissue sarcoma, n/a= not applicable.
